# Microbiota-driven interleukin-17 production provides immune protection against invasive candidiasis

**DOI:** 10.1186/s13054-020-02977-5

**Published:** 2020-05-27

**Authors:** Mengmeng Li, Congya Li, Xianan Wu, Tangtian Chen, Lei Ren, Banglao Xu, Ju Cao

**Affiliations:** 1 Department of Laboratory Medicine, the First Affiliated Hospital of Chongqing Medical University, No.1 Friendship Road, Yuzhong District, Chongqing, 400016 China; 2 Department of Laboratory Medicine, the Third Affiliated Hospital of Chongqing Medical University (Gener Hospital), No.1 Shuanghu Branch Road, Yubei District, Chongqing, 401120 China; 3 Medical Examination Center, the First Affiliated Hospital of Chongqing Medical University, Chongqing, China; 4 Department of Laboratory Medicine, Guangzhou First People’s Hospital, School of Medicine, South China University of Technology, Guangzhou, Guangdong China

**Keywords:** Intestinal microbiota, Invasive candidiasis, IL-17A, Fecal microbiota transplantation, Host immunity

## Abstract

**Background:**

The intestinal microbiota plays a crucial role in human health, which could affect host immunity and the susceptibility to infectious diseases. However, the role of intestinal microbiota in the immunopathology of invasive candidiasis remains unknown.

**Methods:**

In this work, an antibiotic cocktail was used to eliminate the intestinal microbiota of conventional-housed (CNV) C57/BL6 mice, and then both antibiotic-treated (ABX) mice and CNV mice were intravenously infected with *Candida albicans* to investigate their differential responses to infection. Furthermore, fecal microbiota transplantation (FMT) was applied to ABX mice in order to assess its effects on host immunity against invasive candidiasis after restoring the intestinal microbiota, and 16S ribosomal RNA gene sequencing was conducted on fecal samples from both uninfected ABX and CNV group of mice to analyze their microbiomes.

**Results:**

We found that ABX mice displayed significantly increased weight loss, mortality, and organ damage during invasive candidiasis when compared with CNV mice, which could be alleviated by FMT. In addition, the level of IL-17A in ABX mice was significantly lower than that in the CNV group during invasive candidiasis. Treatment with recombinant IL-17A could improve the survival of ABX mice during invasive candidiasis. Besides, the microbial diversity of ABX mice was significantly reduced, and the intestinal microbiota structure of ABX mice was significantly deviated from the CNV mice.

**Conclusions:**

Our data revealed that intestinal microbiota plays a protective role in invasive candidiasis by enhancing IL-17A production in our model system.

## Background

Trillions of microorganisms (including bacteria, archaea, viruses, phages, yeast, and fungi) residing in the gastrointestinal (GI) tract play a vital role in health and disease, and the intestinal microbiota, which has immense impact on nutrition, metabolism, physiology, and immune function of the host, is commonly referred to as a hidden metabolic organ of the body [1–4]. The composition and function of intestinal microbiota in an individual remain stable, and the maintenance of microbiota homeostasis protects host from dysbiosis-related diseases [5]. Intestinal microbiota as an immune modulator plays a pivotal role in the development and maintenance of a healthy immune system of host [6]. The maternal microbiota drives and shapes early postnatal innate immune development [7]; thereafter, an enduring mutualistic partnership developed during long-term host-microbiota interactions [8].

Previous studies using germ-free (GF), antibiotic-treated (ABX), or selectively colonized mice have illustrated that maturation of the immune system depended on intestinal microbiota, while germ-free animals exhibited impaired immune development [9, 10]. Intestinal microbes could regulate the development and function of a variety of immune cells including plasma cells secreting IgA, regulatory cells (Treg cells), T helper cells 17 (Th17 cells), natural killer cells (NK cells), dendritic cells, and mononuclear phagocytes [11]. Alterations in the intestinal microbiota composition and its metabolites were not only linked to gastrointestinal diseases such as inflammatory bowel disease (IBD) [12, 13] and irritable bowel syndrome (IBS) [14], but also associated with obesity [15, 16], nonalcoholic fatty liver disease (NAFLD) [17], insulin sensitivity [18], type 2 diabetes mellitus (T2DM) [19], cancer [20, 21], cardiovascular risk [22, 23], central nervous system disease [24], and allergic disease [25]. There is a growing appreciation of a role for the host-microbiota interactions in human health and disease, as well as the effects of the metabolites and cellular or molecular components on the host immune system [26].

Invasive candidiasis was defined as a group of infectious syndromes resulting from a variety of species of *Candida*, and candidemia is the most commonly recognized syndrome. *Candida* species were main causes of nosocomial bloodstream infections (BSIs); moreover, the major pathogen species of most candidemia cases were *Candida albicans* [27–29]. Besides, recent studies have reported a progressive shift into multidrug-resistant *Candida auris* in the etiology of invasive candidiasis [30, 31]. Invasive candidiasis was associated with prolonged hospital stay in the intensive care unit (ICU), higher healthcare cost, morbidity, and mortality [32, 33]. The risk factors for invasive candidiasis mainly include parenteral nutrition, age, admission to the ICU, organ dysfunction, surgery, immuno-suppression due to chemotherapy and radiotherapy, biofilm formation by *Candida* spp. and antifungal treatment strategies, indwelling central venous catheter (CVC), the usage of assisted ventilation, exposure to broad-spectrum antibiotics, and a gastrointestinal source of candidemia [27, 33–35]. The dysbalance of intestinal microbiota caused by the use of broad-spectrum antibiotics and disruption of mucosal barriers due to surgery were seen as high risk factors for invasive candidiasis, and gastrointestinal colonization was considered as a common source of candidemia, suggesting a role of intestinal microbiota in invasive candidiasis [29, 33, 36].

*Candida albicans* is a normal constituent of human intestinal, and intestinal commensal bacteria maintain immune responsiveness for host against invasive *C. albicans* [37], but the immuneregulatory role of intestinal microbiota in invasive candidiasis is unclear. In this study, we used a mouse model of invasive candidiasis combined with antibiotic cocktail pretreatment to investigate the role of intestinal microbiota in host immunity to invasive candidiasis. Mice after ABX-mediated depletion of intestinal microbiota showed impaired defense during invasive candidiasis. Treatment with rIL-17A or fecal microbiota transplantation (FMT) operation could improve survival of ABX mice after infection. Therefore, intestinal microbiota plays a protective role in invasive candidiasis via regulating IL-17A production.

## Methods

### Mice

Female C57BL/6 mice (6 to 8 weeks old) were purchased from Chongqing Medical University and were maintained in specific pathogen-free (SPF) facilities. These mice were housed in a temperature room with 12-h light-dark cycles and were given free access to autoclaved chow and water. All animal procedures were performed according to the protocol approved by the Institutional Animal Care and Use Committee’s guidelines of the Chongqing Medical University, Chongqing, China.

### Antibiotic treatment

Mice were given autoclaved drinking water supplemented with ampicillin (0.5 mg/mL), gentamicin (0.5 mg/mL), metronidazole (0.5 mg/mL), neomycin sulfate (0.5 mg/mL), vancomycin (0.25 mg/mL), and sucralose (4 mg/mL) as previously described [38, 39]. Antibiotic treatment was started 2–3 weeks prior to infection, and then continued for the duration of the experiment. Antibiotic treatment was withdrawn 24 h prior to fecal microbiota transplantation (FMT) procedure and was replaced with sterile water.

### Systemic infection model of *C. albicans*

*C. albicans* strains SC5314 were cultured in yeast extract, peptone, and dextrose (YPD) medium at 30 °C for 18–24 h [40, 41]. Mice were given an intravenous tail-vein injection of PBS or 2 × 10^5^ colony-forming units (CFUs) *C. albicans*. The weight of each mouse was then daily recorded. Survival was monitored daily for 14 days after *C. albicans* intravenous challenge.

### Quantification of colony-forming units (CFUs)

At day 3 after infection, the livers, kidneys, spleens, and lungs of mice were aseptically dissected, weighed, homogenized, and diluted with PBS, and then serial dilutions of each organ homogenate were plated on YPD agar plates containing penicillin and streptomycin. CFUs were enumerated after incubation for 48 h at 37 °C [42].

### Histopathology

Tissue histology and pathology scores were conducted according to previous studies [43]. Briefly, mice were sacrificed at the designated time points, and the livers, kidneys, spleens, and lungs of mice were harvested, fixed in 4% formalin, embedded in paraffin, sectioned, and stained with hematoxylin and eosin (H&E), and then scored by a pathologist blinded for groups.

For immunohistochemically analysis, kidney sections made from ABX and conventional-housed (CNV) mice at day 3 after infection were formalin-fixed, paraffin-embedded, dewaxed with xylene, and rehydrated in alcohol series. Antigen retrieval, endogenous peroxidase activity blocking, and nonspecific binding site blocking were done before staining. Then sections were incubated with primary antibodies F4/80^+^ or Gr1^+^ (eBioscience) and mouse anti-rat secondary antibody (ZSBIO, Beijing, China), nuclei was stained using hematoxylin, and then color development was conducted by DAB Substrate Kit (ZSBIO). All procedures were done according to the manufacturer’s instructions.

### Serum analysis

Mouse blood was harvested before tissue collection. Then, after centrifugation at 4000 rpm for 10 min at 4 °C, mouse serum was obtained and stored at − 80 °C for further analysis. The serum level of IL-17A were measured using commercially available enzyme-linked immunosorbent assay (ELISA) kits purchased from R&D Systems, while IFN-γ, TNF-α, IL-6, IL-10, IL-12, and IL-22 were assessed by ELISA kits from Neobioscience. The serum concentrations of ALT, AST, BUN, and creatinine of mice were determined by an automatic biochemical analyzer. All procedures were completed according to the manufacturer’s instructions.

### Recombinant IL-17A (rIL-17A) treatment

Mouse rIL-17A protein was purchased from R&D Systems. ABX mice were injected intraperitoneally with a dose of 1 μg rIL-17A protein 8 h before *C. albicans* challenge, followed by a booster dose at 24 h after injection. In parallel, control ABX mice were injected solely with equivalent vehicle.

### Administration of rIFN-γ protein

For in vivo rIFN-γ administration, ABX mice were intravenously injected with 5 μg rIFN-γ protein (R&D Systems) or equivalent vehicle at 8 h before and 24 h after *C. albicans* intravenous challenge.

### Antibody-mediated neutralization of IL-10

We blocked the effects of IL-10 in ABX mice via intravenous injection with 5 μg anti-IL-10 antibodies (R&D Systems) at 2 h before and 24 h after *C. albicans* infection.

### Fecal preparation and transplantation

The procedure of fecal microbiota transplantation was performed as described before [44, 45]. In brief, fresh fecal pellets were collected directly from ten untreated female healthy mice and were pooled, mixed with sterile PBS, and homogenized immediately. The homogenate was centrifuged at 100*g* for 5 min at 4 °C, and the supernatant was used for transplantation. After being switched to regular sterile drinking water, ABX mice in the transplantation group were reconstituted with 200 μl of such suspension by oral gavage 7 days before intravenous challenge, and subsequent 2 days, the reduplicative gavage was conducted, while ABX mice in control group were given 200 μl sterile PBS by the same way. Both groups were intravenously infected with *C. albicans* at day 7 after first gavage operation.

### Fecal bacteria quantification

Fresh feces sampled from uninfected ABX and CNV mice were weighed, homogenized, and serially diluted with PBS, then the dilutions of each sample were plated on blood agar plates in aerobic and anaerobic environments (BBL GasPak Plus system; BD Biosciences) respectively. After incubation for 24 h at 37 °C, CFUs were enumerated and the numbers of fecal bacteria colonies were expressed as CFUs/g feces.

### DNA extraction and 16S ribosomal RNA gene sequencing

Fresh fecal pellets (about 150 mg per mouse) were collected from non-infected ABX and CNV mice and frozen at − 80 °C within 2 h after sampling until analysis. Microbial DNA was extracted using the E.Z.N.A.® Soil DNA Kit (Omega Bio-tek, Norcross, GA, USA), following the standard procedures [46]. The V3-V4 hypervariable regions of the bacteria’s 16S rRNA genes were amplified with barcoded primers 338F (5′-ACTCCTACGGGAGGCAGCAG-3′) and 806R (5′-GGACTA-CHVGGGTWTCTAAT-3′). The purified amplicons were pooled in equimolar and paired-end sequenced (2 × 300) on an Illumina MiSeq platform (Illumina, San Diego, USA) [47].

### Microbial analysis

The sequencing raw reads were demultiplexed and filtered using QIIME (version 1.9.1) [48]. Operational taxonomic units (OTUs) were clustered with 97% similarity cutoff by UPARSE (version 7.1), and chimeric sequences were identified and removed by UCHIME [49]. The taxonomy of each 16S rRNA gene sequence was analyzed by RDP Classifier algorithm against the Silva (SSU128) 16S rRNA database using confidence threshold of 70% [50]. Rarefaction analysis and alpha-diversity calculations were performed on the OTU table, and beta-diversity was measured by computing unweighted UniFrac and was visualized by principal coordinate analysis (PCoA) according to the matrix of distance [51]. The linear discriminant analysis (LDA) effect size (LEfSe) was performed using LEfSe program, and an effect size threshold (on the logarithmic LDA score) of 2.0 was used [52].

### Statistical analysis

Data were expressed as the mean ± SEM. Statistical analyses were performed using GraphPad Prism 7.0 or SPSS 21.0 software. Details of individual tests are included in the figure legends. Comparisons between two groups of data were analyzed by Mann-Whitney *U* test. The weight loss over time after infection in comparison to starting weight between groups was analyzed using two-way ANOVA. Survival analysis over time after infection was assessed by the Kaplan-Meier analysis followed by log-rank tests. A two-tailed *P* values less than 0.05 was considered statistically significant.

## Results

### Depletion of intestinal microbiota decreased survival in a murine model of invasive candidiasis

**Fig. 1:**
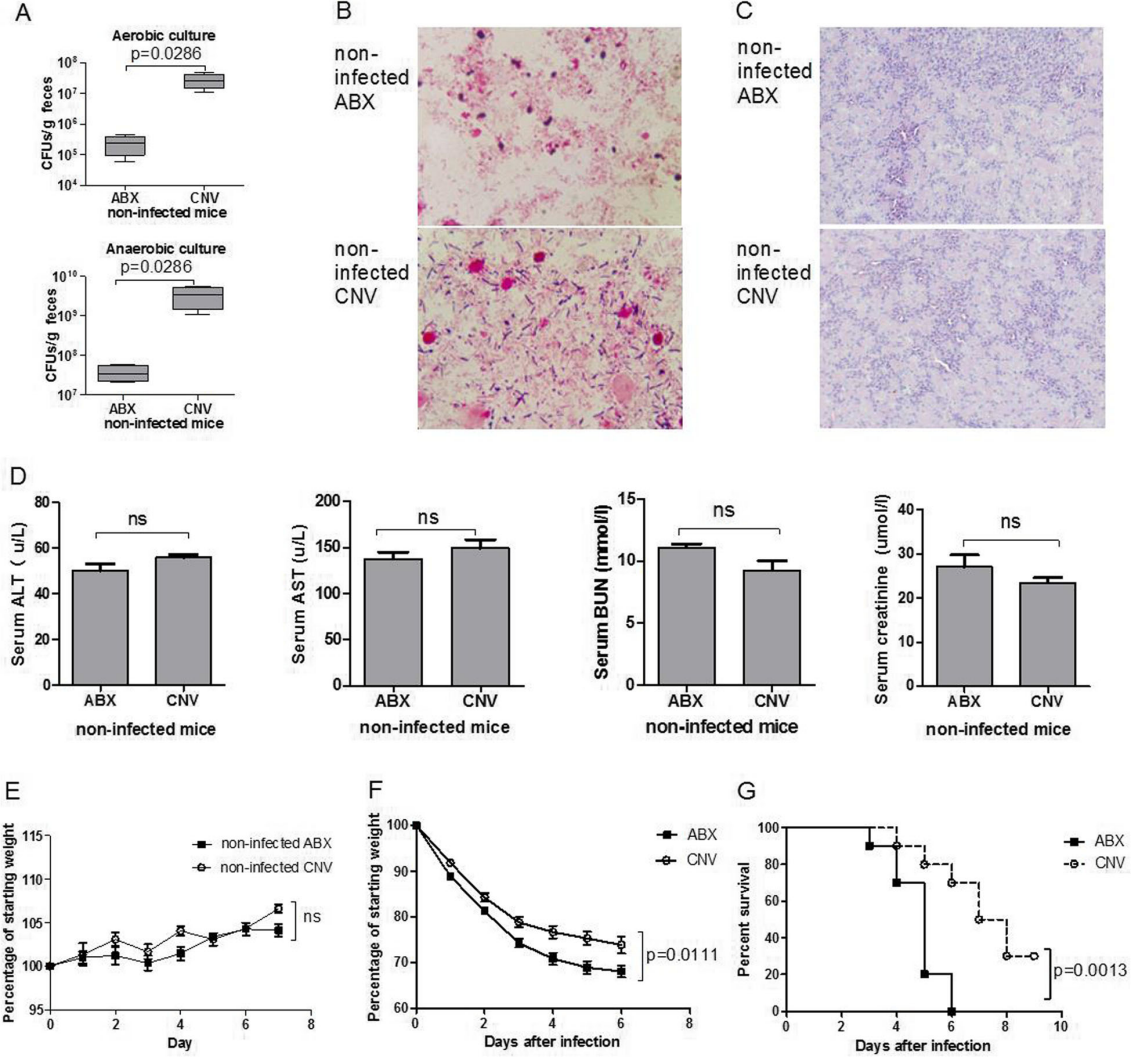
Decreased survival ability of ABX mice in invasive candidiasis. **a** Fecal bacteria quantification of non-infected ABX and CNV mice in aerobic and anaerobic environments (*n* = 4). **b** Feces smear stained using Gram stain from non-infected ABX and CNV mice. **c** Representative examples of H&E-stained kidney from non-infected ABX and CNV mice. **d** The serum concentrations of ALT, AST, BUN, and creatinine of non-infected ABX and CNV mice. **e** Weight curve of non-infected ABX and CNV mice. **f** Body weight loss of ABX and CNV mice were daily monitored after intravenous injection with fungal yeasts (2 × 105 CFUs *C. albicans* strain SC5314) and depicted with plot (*n* = 6). **g** Survival analysis of the ABX and CNV mice after injection (*n* = 10). Data were expressed as mean ± SEM, and *P* values were analyzed by Mann-Whitney *U* test (**a**, **d**), two-way ANOVA (**c**), and log-rank test (**d**). Two-tailed *P* values < 0.05 were considered statistically significant. Results are representative of three independent experiments

To investigate the role of the intestinal microbiota in invasive candidiasis, we first treated SPF C57BL/6 mice with antibiotic cocktail for 2–3 weeks and then these mice were intravenously challenged with *C. albicans*. A previous study has confirmed the validity of this approach to deplete intestinal microbiota [38]. In parallel, CNV mice were challenged with an equivalent amount of candida yeasts. In our model, the total population of cultivable bacteria was significantly reduced in non-infected ABX mice as demonstrated by fecal bacterial quantification under both aerobic and anaerobic environments when compared with non-infected CNV mice (Fig. 1a), and numbers of both gram-positive and gram-negative bacteria were dramatically decreased in non-infected ABX mice as shown in feces smear stained using Gram stain (Fig. 1b). Fungal culture of feces from both non-infected ABX and CNV mice showed that the intestinal colonization of fungi relatively increased after the antibiotic cocktail treatment which is in accordance with previously reported studies (Figure S1A). However, fungal colony was not detected in blood and kidney of both non-infected ABX and CNV mice (Figure S1B-C), and there was no pathological damage in the kidney of both non-infected ABX and CNV mice as showed in H&E-stained kidney sections (Fig. 1c). The serum concentrations of ALT and AST (markers for hepatocellular injury), BUN, and creatinine (markers for renal dysfunction) were not significantly different in non-infected ABX and CNV mice (Fig. 1d). There was no significant difference in the weight gain curves between non-infected ABX and CNV mice (Fig. 1e). In addition, adult healthy mice were resistant to *C. albicans* GI colonization, and we determined that there was no *Candida albicans* colonized in the GI of conventional C57BL/6 mice and non-infected ABX mice which maintained in SPF facilities during current breeding environment by using Candida chromogenic plate (Autobio) combining with mass spectrometer, and the *Candida slooffiae* might be the main member of gut fungal communities in our mice. These data thus showed that antibiotic cocktail treatment depleted intestinal microbiota in mice without causing systemic fungal infection and tissue damage despite the relatively increased intestinal colonization of fungi (non-*C. albicans*).

After intravenous challenge with *C. albicans*, both ABX and CNV mice were in a poor state and unanimated, indicating a systemic infection condition in mice. ABX mice showed significantly higher weight loss (Fig. 1f) and mortality (Fig. 1g) when compared with CNV mice. These results indicated that mice had impaired survival ability against invasive candidiasis after depletion of commensal microbiota.

### Depletion of intestinal microbiota impaired host defense during invasive candidiasis

**Fig. 2:**
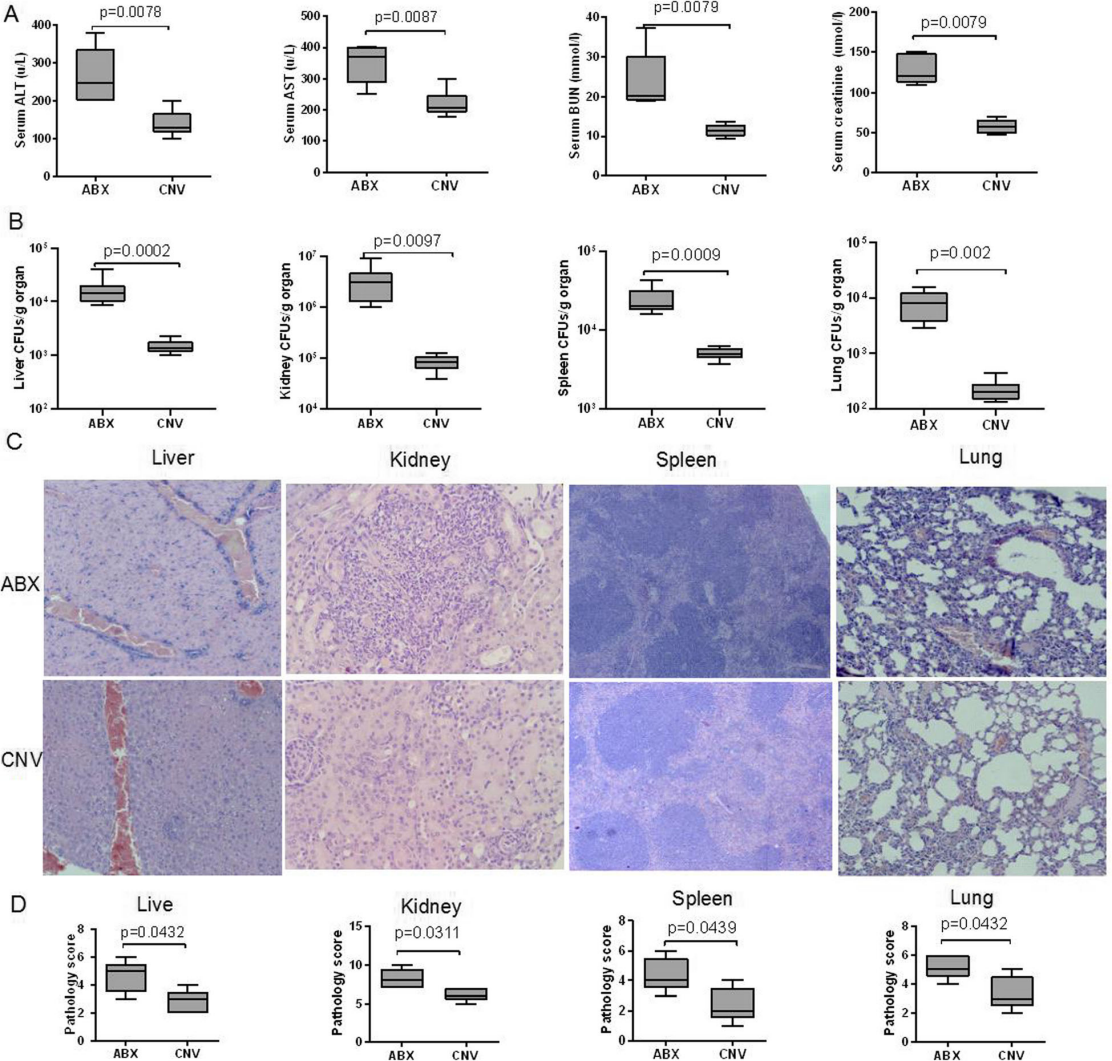
Impaired defense against invasive candidiasis in ABX mice. All samples were obtained from ABX and CNV mice at day 3 after infection. **a** The serum concentrations of ALT, AST, BUN, and creatinine of ABX and CNV mice. **b** Fungal burden of the liver, kidney, spleen, and lung tissues from ABX and CNV mice. **c** Representative examples of H&E-stained liver, kidney, spleen, and lung from ABX and CNV mice. **d** Pathological scores for liver, kidney, spleen, and lung tissues from ABX and CNV mice. Data were expressed as mean ± SEM, and *P* values were analyzed by Mann-Whitney *U* test (**a**, **b**, **d**). Two-tailed *P* values < 0.05 were considered statistically significant. Results are representative of three independent experiments (*n* = 5, in each group)

Invasive candidiasis-associated mortality was closely related to multiple organ damages. Serum concentrations of ALT, AST, BUN, and creatinine were significantly higher in ABX mice than those in CNV mice at day 3 after infection (Fig. 2a). The fungal burdens in the livers, kidneys, spleens, and lungs of ABX mice were also significantly higher than those of CNV mice (Fig. 2b), suggesting the decreased ability to eliminate fungal pathogens in host. The fungal invasion led to more severe histopathological injury in ABX mice (Fig. 2c, d). In addition, the weight of livers, spleens, and lungs in ABX mice were significantly lighter than those in CNV mice, but the kidney was not (Figure S2A-E). Kidney injury was further validated by kidney sections stained with periodic acid Schiff (PAS) (Figure S2F). These data therefore suggest that the kidney is the primary target organ in the murine model of *C. albicans* infection, which was in accord with the data from previous studies [42, 53]. We then mainly focused on the kidney injury to determine the infection extent during invasive candidiasis in the later animal experiment.

### Inflammatory response was aggravated in ABX mice during invasive candidiasis

**Fig. 3:**
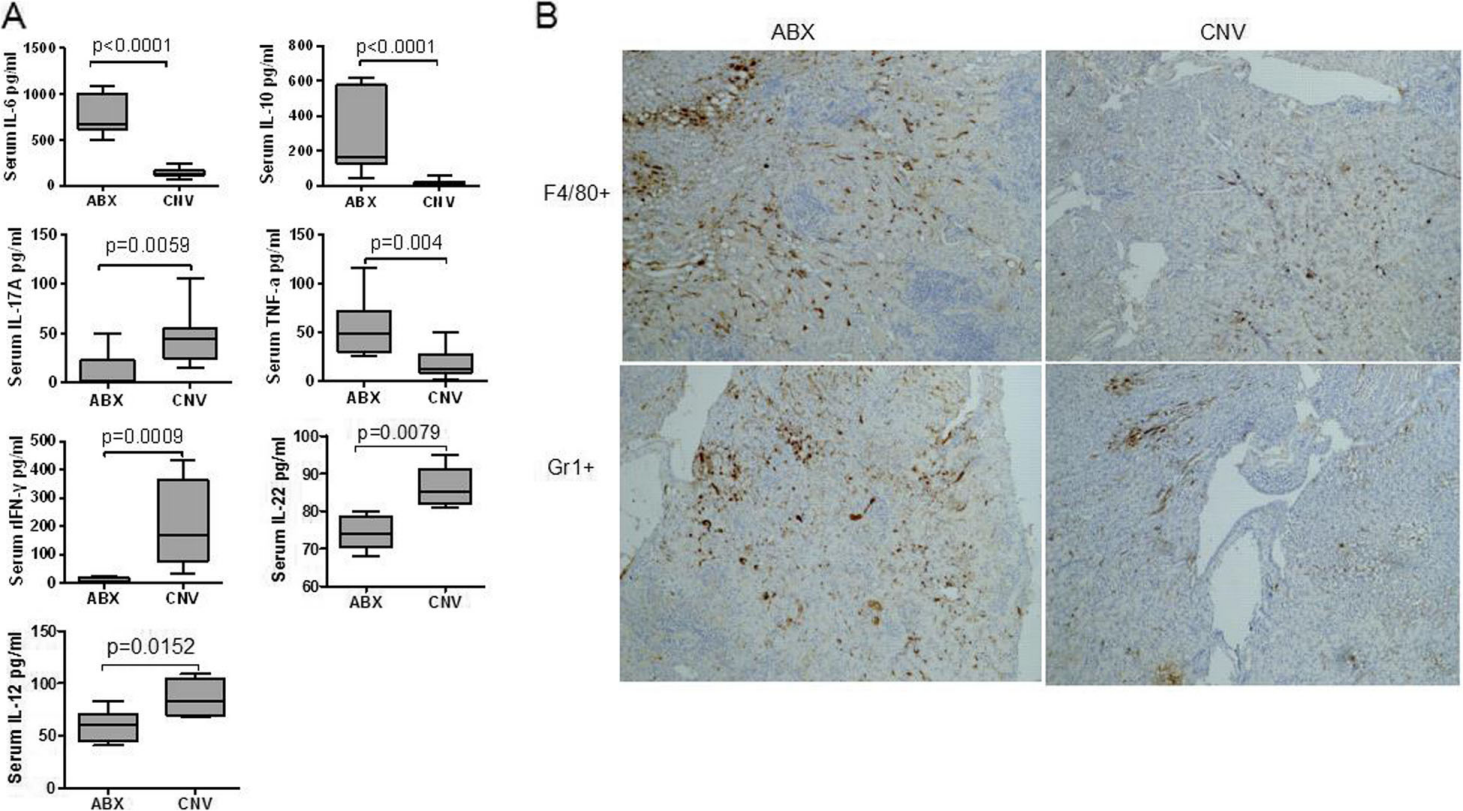
Changes of inflammatory response in ABX and CNV mice. Samples were obtained from ABX and CNV mice at day 3 after infection. **a** Serum concentrations of IL-6, IL-10, IL-17A, TNF-α, IFN-γ, IL-12, and IL-22 in ABX and CNV mice (*n* = 9). Data were expressed as mean ± SEM, and *P* values were analyzed by Mann-Whitney *U* test. Two-tailed *P* values < 0.05 were considered statistically significant. Results are representative of three independent experiments. **b** Representative images of immunohistochemical staining to determine the infiltration of macrophages (F4/80^+^) and neutrophils (Gr1^+^) in kidney tissues

Considering the modulatory effects of commensal microbiota on host immunity, we then measured inflammatory cytokine levels at day 3 after *C. albicans* infection. As shown in Fig. 3a, the concentrations of serum IL-6, IL-10, and TNF-α in ABX group were significantly higher than those in the CNV group, whereas the levels of IL-17A, IFN-γ, IL-12, and IL-22 were significantly decreased. A previous study has shown that administration of IL-10 increased host susceptibility to candidiasis [54], while IL-17, IFN-γ, IL-12, and IL-22 played an important role in protecting against *candida* [55].

The infiltration degree of inflammatory cells (macrophages and neutrophils) of the renal tissues in ABX group was also significantly higher than that in CNV group (Fig. 3b). In addition, there were no significant differences in the function (killing capacity to *C. albicans* spores) of peritoneal macrophages, peritoneal neutrophils, and blood neutrophils despite the number of peritoneal macrophage in non-infected ABX mice was lower than that in non-infected CNV mice (Figure S3), indicating that depleting intestinal microbiota did not affect the killing capacity of macrophages and neutrophils to *C. albicans* spores. Therefore, depleting intestinal microbiota caused aggravated inflammatory response during invasive candidiasis.

### IL-17A administration provided protection against invasive candidiasis in ABX mice

**Fig. 4:**
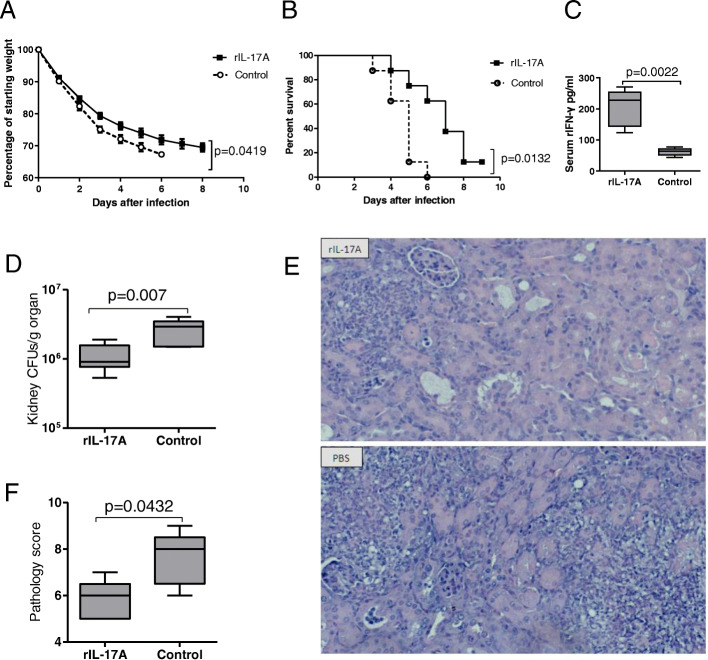
Protection provided by administration of rIL-17A for ABX mice during invasive candidiasis. **a** Plot of weight loss over time after infection in comparison to starting weight for rIL-17A treatment group and control group (*n* = 5). **b** Survival analysis of both groups after infection (*n* = 8). **c**–**f** Serum concentrations of IFN-γ (**c**) (*n* = 6), fungal burden of the kidneys (**d**) (*n* = 7), representative image of H&E-stained kidney (**e**) and corresponding pathological scores (**f**) (*n* = 5) in mice from both rIL-17A treatment group and control group at day 3 after infection. Data were expressed as mean ± SEM. *P* values were analyzed by two-way ANOVA (**a**), log-rank test (**b**), and Mann-Whitney *U* test (**c**, **d**, **f**). Two-tailed *P* values < 0.05 were considered statistically significant. Results are representative of three independent experiments

To figure out the role of cytokines in invasive candidiasis after depletion of commensal microbiota, ABX mice were administered with recombinant cytokines or blocking antibodies. Compared with control group, rIL-17A administration significantly improved weight loss (Fig. 4a), decreased mortality (Fig. 4b), upregulated IFN-γ production (Fig. 4c), decreased renal fungal burden (Fig. 4d), and alleviated kidney injury (Fig. 4e, f) in ABX mice after *C. albicans* infection. However, there was no significant difference in weight loss and survival rate between the rIFN-γ-treated and control-treated ABX mice (Figure S4). In addition, blocking the effect of IL-10 could not alleviate the infectious condition of ABX mice (Figure S5). The protective contribution of IL-17 to *Candida* infection has been shown by a previous study [32], and our data also suggested that IL-17 was actively involved in exerting protective effect by intestinal microbiota on invasive candidiasis.

### FMT could alleviate invasive candidiasis in ABX mice

**Fig. 5:**
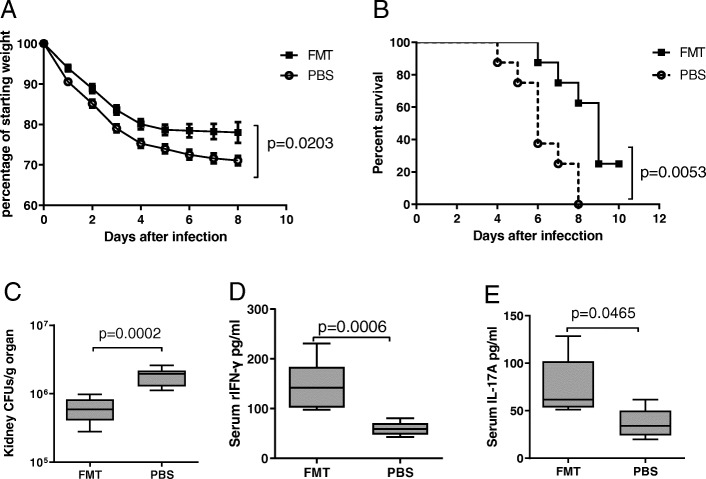
FMT alleviated morbid conditions of ABX mice in invasive candidiasis. **a**, **b** Plot of weight loss (**a**) over time after infection in comparison to starting weight (*n* = 5) and survival analysis (**b**) (*n* = 8) for both FMT group and PBS group at day 3 after infection. **c**–**e** Fungal burden of the kidneys (**c**) (*n* = 8) and serum concentration of IFN-γ (**d**) (*n* = 7) and IL-17A (**e**) (*n* = 5) in mice from FMT group and PBS group at day 3 after infection. Data were expressed as mean ± SEM. *P* values were analyzed by two-way ANOVA (**a**), log-rank test (**b**), and Mann-Whitney *U* test (**c**–**e**). Two-tailed *P* values < 0.05 were considered statistically significant. Results are representative of three independent experiments

We further restored the intestinal microbiota of ABX mice by transplanting fresh feces from healthy SPF C57BL/6 mice. FMT was able to improve weight loss (Fig. 5a) and mortality (Fig. 5b) and to decrease fungal burden in the kidney (Fig. 5c). Furthermore, FMT could increase the serum level of IFN-γ and IL-17A (Fig. 5d) in ABX mice during invasive candidiasis.

### Overall intestinal microbiota structure was changed in ABX mice

**Fig. 6:**
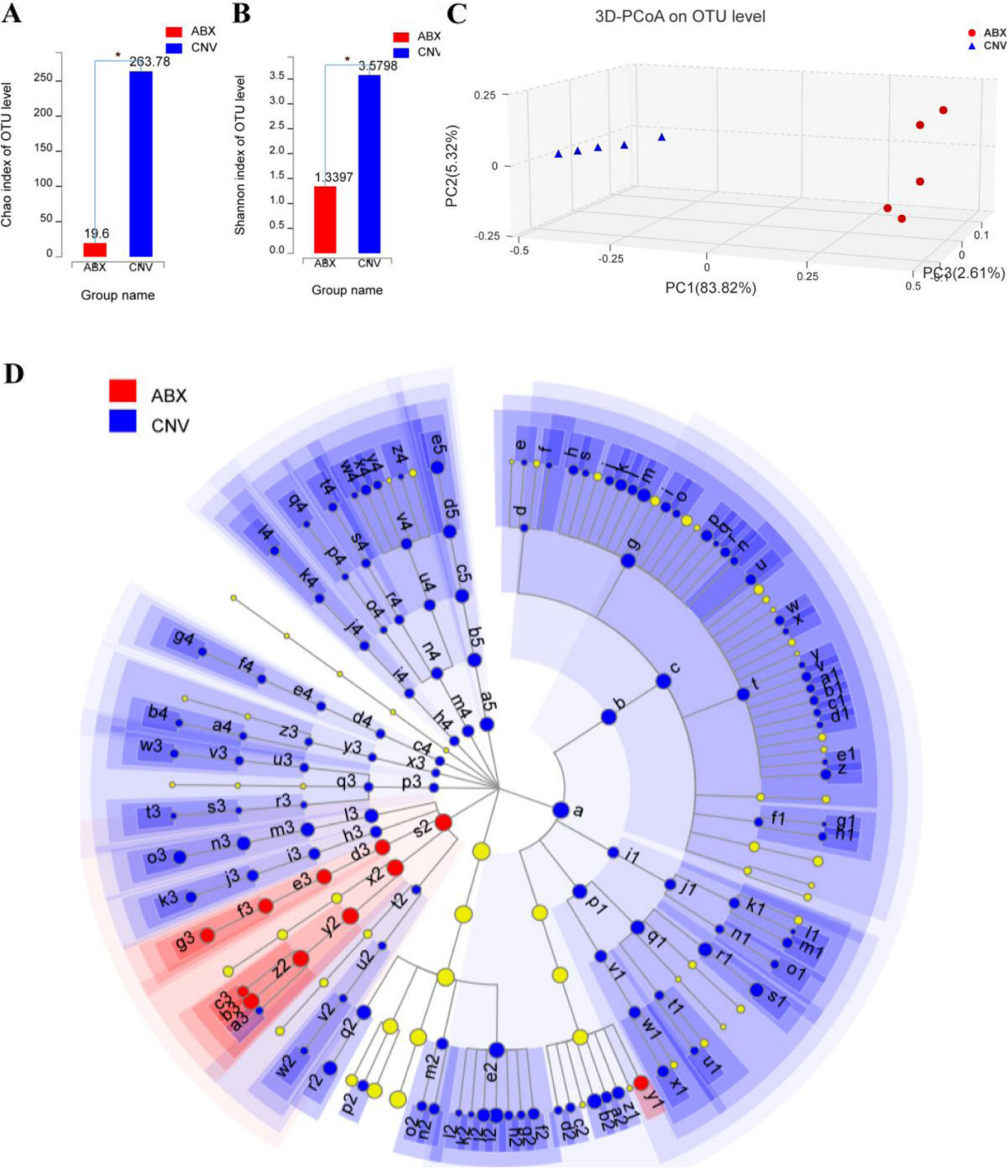
The intestinal microbiota structure of ABX mice significantly deviated from CNV mice. (*n* = 5). **a**, **b** Comparison of alpha diversity in Chao (**a**) and Shannon (**b**) index of ABX and CNV mice performed on the OTU tables (*p* = 0.012 and *p* = 0.012, Wilcoxon rank-sum test). **c** Principal coordinate analysis (PCoA) of unweighted UniFrac analysis on OTU level (*p* = 0.009, PERMANOVAR). **d** Differences in cladogram of different microbiota taxa from the phylum level down to the genus level were represented by the color of the most abundant class. The color of red, green, and yellow respectively indicate taxa enriched in the ABX and CNV groups and nonsignificant taxa, and the diameter of each circle is proportional to the taxon’s abundance

**Fig. 7:**
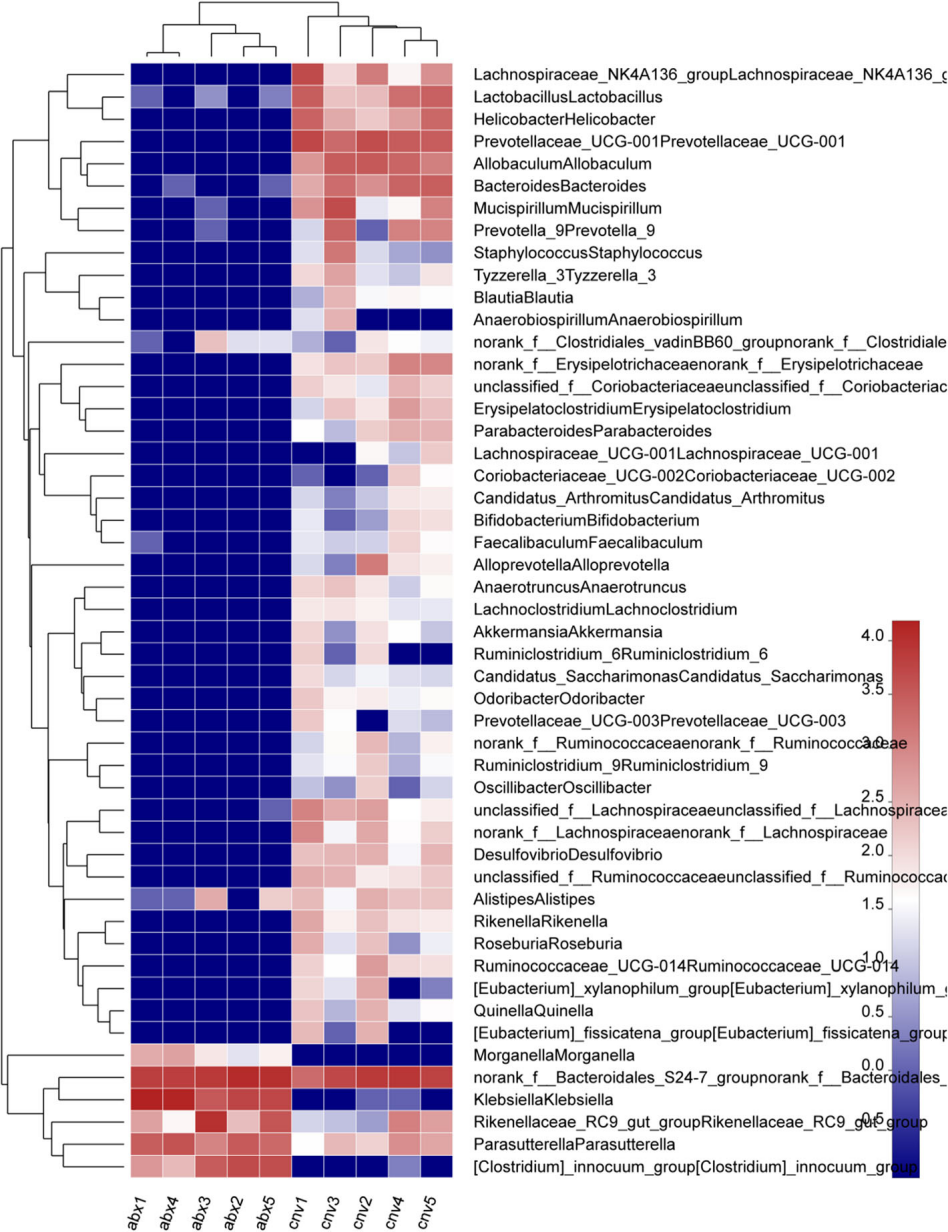
Heat map of genus in ABX and CNV mice. Heat map of the genus in the top 50 of the total abundance at the classification level (*n* = 5). The color of the spots in the right panel represents the mean relative abundance in each sample (the color of blue, white, and red respectively represents less abundance, intermediate abundance, and the most abundance). The *x*-axis and *y*-axis is a sample clustering tree and species clustering tree

The intestinal microbiota structure of non-infected ABX and CNV mice were profiled through 16S rRNA gene sequencing analysis by Illumina MiSeq platform using fecal samples. After raw reads filtering, 50,795 sequences per sample in average were obtained (min 34,570; max 66,272). The coverage index (more than 99% in each sample) showed that these sequences could represent the majority of bacteria in each sample and the rarefaction curve demonstrated the adequate sequencing depth (Figure S6A-B). The significant differences of Chao and Shannon indices indicated that the community richness and community diversity in non-infected ABX mice were lower than those in non-infected CNV mice (Fig. 6a, b). As for beta-diversity, an unweighted UniFrac-based principal coordinated analysis (PCoA) according to OTUs of each sample was performed to visualize the main variations, and a significantly separate clustering of the intestinal microbiota structure was observed between the two groups (Fig. 6c). The overlap between non-infected ABX and CNV group at the genus level was depicted in a Venn diagram (Figure S6C), and the microbiota composition at the phylum level and genus level in each group was showed in Figure S7. A cladogram representation of taxonomy-based comparisons showed the significant structure from the phylum level down to the genus level of intestinal microbiota between the non-infected ABX and CNV group as determined by LEfSe (Fig. 6d). The community heat map depicted dominant genus in the top 50 of the total abundance at the classification level in two groups (Fig. 7). According to the above data, the dominant phyla of both non-infected ABX and CNV group were *Bacteroidetes* (44.76% vs 52.47%), *Proteobacteria* (44.76% vs 7.10%), and *Firmicutes* (10.48% vs 33.77%), and notably, there were hardly any bacteria in the non-infected ABX group which belong to other seven phyla (*Deferribacteres*, *Actinobacteria*, *Tenericutes*, *Cyanobacteria*, *Verrucomicrobia*, *Spirochaetae*, and *Saccharibacteria*). Compared to the non-infected CNV group, just the *Proteobacteria* were enriched while the other phyla were markedly reduced in the non-infected ABX mice. At the genus level, the genera of *Klebsiella*, *Parasutterella*, *Clostridium innocuum* group, and *Morganella* were significantly enriched in non-infected ABX group (in order of abundance). The statistically discrepant genera ranked by the front in order of abundance in the non-infected CNV group were *Prevotellaceae-UCG-001*, *Allobaculum*, *Bacteroides*, *Lactobacillus*, *Lachnospiraceae NK4A136* group, *Mucispirillum*, *Helicobacter*, and *Prevotella-9*. *Parasutterella* has been reported to be associated with chronic intestinal inflammation in irritable bowel syndrome patients [56]. *Parasutterella* remarkably increased in the non-infected ABX mice may be related to the defective defense against invasive candidiasis. *Lachnospiraceae* abundance was decreased in a model of ulcerative colitis [57]. *Lactobacillus* could reduce the virulence of *C. albicans* [58], and its metabolites inhibit *C. albicans* biofilm formation [59]. In summary, there were significant changes in intestinal microbiota structure between ABX and CNV mice, and the significant discrepant bacteria may play a key role during invasive candidiasis in mice and further study is required to explore the specific bacteria which actually work during invasive candidiasis.

## Discussion

In the recent years, the role of intestinal microbiota in host immunity and human disease has gained more and more attention. Intestinal microbiota has an effect on the development of the immune system and the susceptibility to infectious diseases [60]. In this study, we demonstrated a protective role for intestinal microbiota in host resistance against invasive candidiasis. Mice after depletion of intestinal microbiota exhibited impaired defense against invasive candidiasis, expressed as significantly increased weight loss, mortality, tissue fungal burden, and tissue damage, compared with the CNV mice. The serum level of IL-17A in ABX mice was significantly decreased after infection, while IL-17A played a protective role in the resistance to *C. albicans* infection [61]. IL-17A treatment could improve survival of ABX mice after invasive candidiasis. However, as aberrant IL-17 could lead to systemic inflammation as well as autoinflammatory conditions, the potential harms should be concerned when rIL-17A was used as a potential therapy, which requires further study.

Previous studies have shown that commensal bacteria could affect the function of host immune cells. For example, macrophages from ABX mice exhibited decreased expression of genes associated with antiviral immunity, defective responses to type I and type II IFNs, and impaired capacity to limit viral replication [39]. Effective host defense against fungi required Th1- and Th17-mediated immunity, whereas Th2 type responses were generally associated with adverse outcomes, and Th17 responses have been shown to be even more important than Th1 cell responses in antifungal immunity [55]. Our study found that ABX mice exhibited a decreased Th1 type T cell responses (indicated by decreased IFN-γ) and Th17 cell response (indicated by decreased IL-17A and IL-22) and increased Th2-type responses (indicated by increased IL-10 and IL-6). Intestinal microbiota exerted protective effect through regulating IL-17 production during invasive candidiasis.

Restoring the intestinal microbiota by FMT could enhance the defense ability and the expression of IL-17A in ABX mice against invasive candidiasis. In addition, the intestinal microbiota diversity of ABX mice was significantly reduced, and the intestinal microbiota structure of ABX mice was significantly deviated from the CNV mice. The complicated interactions between intestinal microbiota and host immune system play a key role in controlling gut barrier [62]. Gut microbes were recognized and monitored by the innate immune system with pattern recognition receptors (PRRs), a kind of recognition molecule which could recognize one or more pathogen-associated molecular patterns (PAMPs), such as toll-like receptors (TLRs), NOD-like receptors (NLRs), and C-type lectin receptors (dectin-1, dectin-2) [63]. TLR-2 is vital for murine defense against *C. albicans* [64]; *Lactobacillus crispatus* could promote epithelial cell defense against *C. albicans* via TLR-2 and TLR-4 [65]. Dectin-1 mediates recognition of *C. albicans* [66]. Dectin-1 could modify microbiota to regulate the homeostasis of intestinal immunity [67]. Dectin-1 could recognize β-(1,3)-glucan and mediate downstream Syk kinase signaling resulting in the secretion of IL-6 and IL-23, while IL-6 and IL-23 could promote IL-17 expression in T cells [68]. Therefore, the immune communication is essential for intestinal microbiota to protect against invasive candidiasis. A previous study has indicated that commensal bacteria eradication enhanced protection against disseminated *C. albicans* infection [38], which is in contrast with our present work. The following two points may explain this difference. Firstly, different strain types (standard vs recombinant) and inoculation dosage (2 × 10^5^ VS 5 × 10^4^ CFUs) of SC5314 were used in two studies. Secondly, variations in intestinal microbiota of two studies may result in a different phenotype due to confounding factors in the experimental setup, such as maternal effects, cage effects, mouse vendors, and housing conditions (diet, light, stress factors) [69]. Moreover, some studies have shown that intestinal commensal microbiota could provide protective effects on host defense [70, 71] and prevent *C. albicans* colonization and dissemination from the gut [72]. Besides, the number of anaerobic bacteria and abundance of Lactobacillus were all significantly reduced in ABX mice. However, a decrease in anaerobic bacteria could promote overgrowth of *Candida glabrata* [73], *Lactobacillus crispatus* could modulate epithelial cell defense against *C. albicans* [65], clinical strains of *Lactobacillus* could influence the growth and expression of *C. albicans* virulence factors [58], and the metabolites of *L. gasseri* and *L. crispatus* could downregulate biofilm formation-related genes of *C. albicans*, thus inhibiting biofilm formation of *C. albicans* [59]. These previous studies suggest that we should explore in detail the specific mechanisms by which members of the intestinal microbiota communicate with the host immune system and identify specific species as different members of intestinal microbiota elicit different immune responses relating to itself or its metabolites. In view of different individuals having different structures of intestinal microbiota, so do mice and humans, pinpointing the immune response initiated by specific bacteria could help to apply the findings into human research. Given this, we characterized the microbiota structure and discrepant genera of non-infected ABX and CNV mice in the intestine.

In addition, numerous metabolites produced by the microbiota affect host metabolism mostly by combining with specific membranes or nuclear receptors of host [74] and act as extracellular signaling molecules to activate cell-surface G-protein-coupled receptors (GPCRs) [75]. Notably, except for the role of intestinal microbiota in the development and activation of the host immune system, the adaptive immunity also in turn has a predominant effect on regulating gut microbiota’s composition and diversity [76]. Thereby, the pivotal communications network between intestinal microbiota and host immune are considerably intricate and delicate. Hence, investigation for the specific role in host immune of bacteria and its metabolites in intestine will be an enormous but a worthwhile task.

There are a few important limitations to this study. Firstly, antibiotic treatment in mouse model could cause alterations in the gut including depletion of the microbiota, direct effects of antibiotics on host tissues, and the effects of remaining antibiotic-resistant microbes [77], whose impact on our study is difficult to estimate. Although our model has shown that the routine biochemical indicators, weight curve, and renal pathology of ABX mice were consistent with CNV mice, future studies with a shorter duration of antibiotics are still needed to be performed. Secondly, as the intravenous inoculation of Candida might likely not as clinically relevant as gut colonization preceding systemic infection, further study using mouse model of invasive candidiasis via GI *C. albicans* colonization is needed. Finally, this work depicted the influence of intestinal microbiota on invasive candidiasis, while how the intestinal microbiota communicates with the cytokines in circulatory system during invasive candidiasis remains to be elucidated.

## Conclusions

In conclusion, this study characterized the protective role of intestinal microbiota in limiting immunopathology and improving survival ability of host against invasive candidiasis, and we revealed that IL-17 plays an important role in intestinal microbiota-mediated protection against invasive candidiasis. Therefore, these data enlarge our understanding of intestinal microbiota, host immunity, and human diseases. Future studies are required to figure out the mechanisms involved in microbiota-mediated protection against invasive candidiasis, such as the location and function of Th17 cells, the pathway by which intestinal microbiota communicates with IL-17A and other factors (IFN-γ, IL-12, IL-22), the interaction between these cytokines, and the verification of specific species working during invasive candidiasis.

## Supplementary information

**Additional file 1: Figure S1.** Fungal culture of feces, blood and kidney from non-infected ABX and CNV mice. (A-C) Fungal culture of feces (A), blood (B) and kidney (C) from non-infected ABX and CNV mice. Data were expressed as mean ± SEM. *P* values were analyzed by Mann-Whitney U test (A-D). Two-tailed P values <0.05 were considered statistically significant. N.D. means not detected.

**Additional file 2: Figure S2.** Weight of organs and kidney histology in ABX and CNV mice. (A-D) Weight of livers, kidneys, spleens and lungs tissues at day 3 after infection. (E) Picture of size comparison in mice kidney. (F) Kidney sections stained with periodic acid Schiff (PAS) from ABX and CNV mice. *P* values were assessed by Mann-Whitney U test (A-D). Two-tailed P values <0.05 were considered statistically significant.

**Additional file 3: Figure S3.** The number and function of macrophages and neutrophils. (A) The number of peritoneal macrophages. (B) The phagocytosis of peritoneal macrophages to *C. albicans* spores. (C-D) the killing capacity to *C. albicans* spores of peritoneal neutrophils (C) and blood neutrophils (D). (E) Immunofluorescence staining showed the phagocytosis of macrophages in ABX group (E1) and CNV group (E2) (*P*>0.05). Data were expressed as mean ± SEM. P values were analyzed by Mann-Whitney U test (A-D). Two-tailed P values <0.05 were considered statistically significant.

**Additional file 4: Figure S4.** Administration of rIFN-γ. (A-B) Weight loss (A) over time after infection in comparison to starting weight and survival analysis (B) for both the IFN-γ treatment group and control group. P value was analyzed by two-way ANOVA (A) (*p*>0.05) and log-rank test (B) (p>0.05).

**Additional file 5: Figure S5.** Antibody-mediated neutralization of IL-10. (A) Weight loss of both the IL-10 blocking group and control group after infection was analyzed by two-way ANOVA (P>0.05). (B) Survival analysis was assessed using log-rank test (*P*=0.0172). Two-tailed P values <0.05 were considered statistically significant.

**Additional file 6: Figure S6.** Microbial diversity index and Venn diagram. (A) The coverage index. (B) The rarefaction curve of OTU levels for all samples based on the number of sequences drawn and the sobs index of OTU level. (C) Venn diagram of the overlap in genus level.

**Additional file 7: Figure S7.** Community bar-plot and Wilcoxon rank-sum test bar plot. (A-B) Community bar-plot analysis shows relative abundance of intestinal microbiota in each sample at the phylum level (A) and genus level (B). (C-D) Wilcoxon rank-sum test bar plot on the phylum level (C) and genus level (D) showed significant genus in the top 20 of the total abundance at the classification level (*n* = 5, in each group).

## Data Availability

The datasets supporting the conclusions of this article are included within the article and its additional files. Raw 16S rRNA gene sequence data is available through NCBI’s SRA database under the accession number SRP229663 (BioProject: PRJNA589247).

## Abbreviations

ABX: Antibiotic-treated
CNV: Conventional-housed
Th17 cell: T helper cell 17
IL-17A: Interleukin-17A
FMT: Fecal microbiota transplantation
SPF: Specific pathogen-free
ELISA: Enzyme-linked immunosorbent assay
PBS: Phosphate-buffered saline
IFN-γ: Interferon-gamma
TNF-α: Tumor necrosis factor-alpha
ALT: Alanine aminotransferase
AST: Aspartate aminotransferase
BUN: Blood urea nitrogen
CFUs: Colony-forming units
LEfSe: Linear discriminant analysis (LDA) effect size
GI: Gastrointestinal
