# Supplementary figures and images for: Microbiota-driven interleukin-17 production provides immune protection against invasive candidiasis
Source: Crit Care. 2020 May 27;24:268. doi: 10.1186/s13054-020-02977-5 (PMC7251893; doi:10.1186/s13054-020-02977-5)

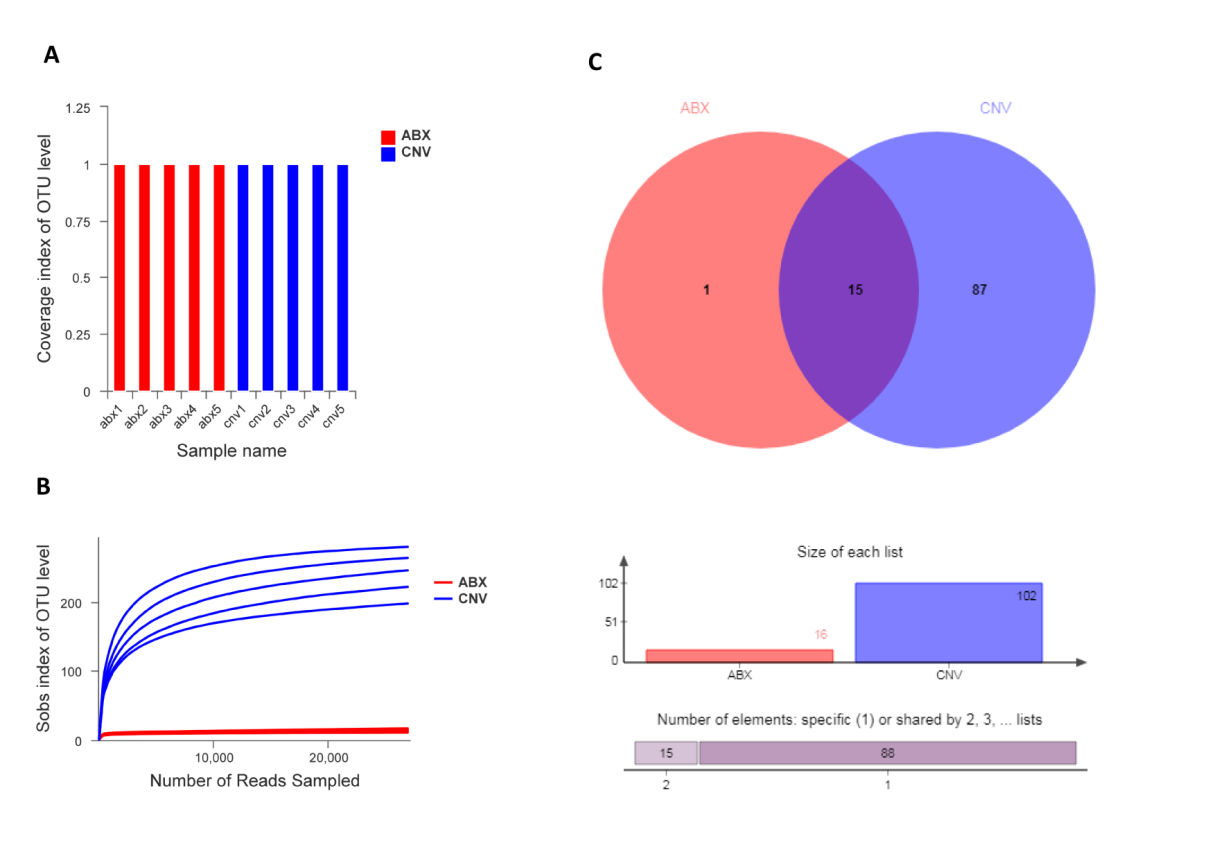

Supplement: Supplementary file 6 — Additional file 6: Figure S6. Microbial diversity index and Venn diagram. (A) The coverage index. (B) The rarefaction curve of OTU levels for all samples based on the number of sequences drawn and the sobs index of OTU level. (C) Venn diagram of the overlap in genus level. [file 13054_2020_2977_MOESM6_ESM.docx]

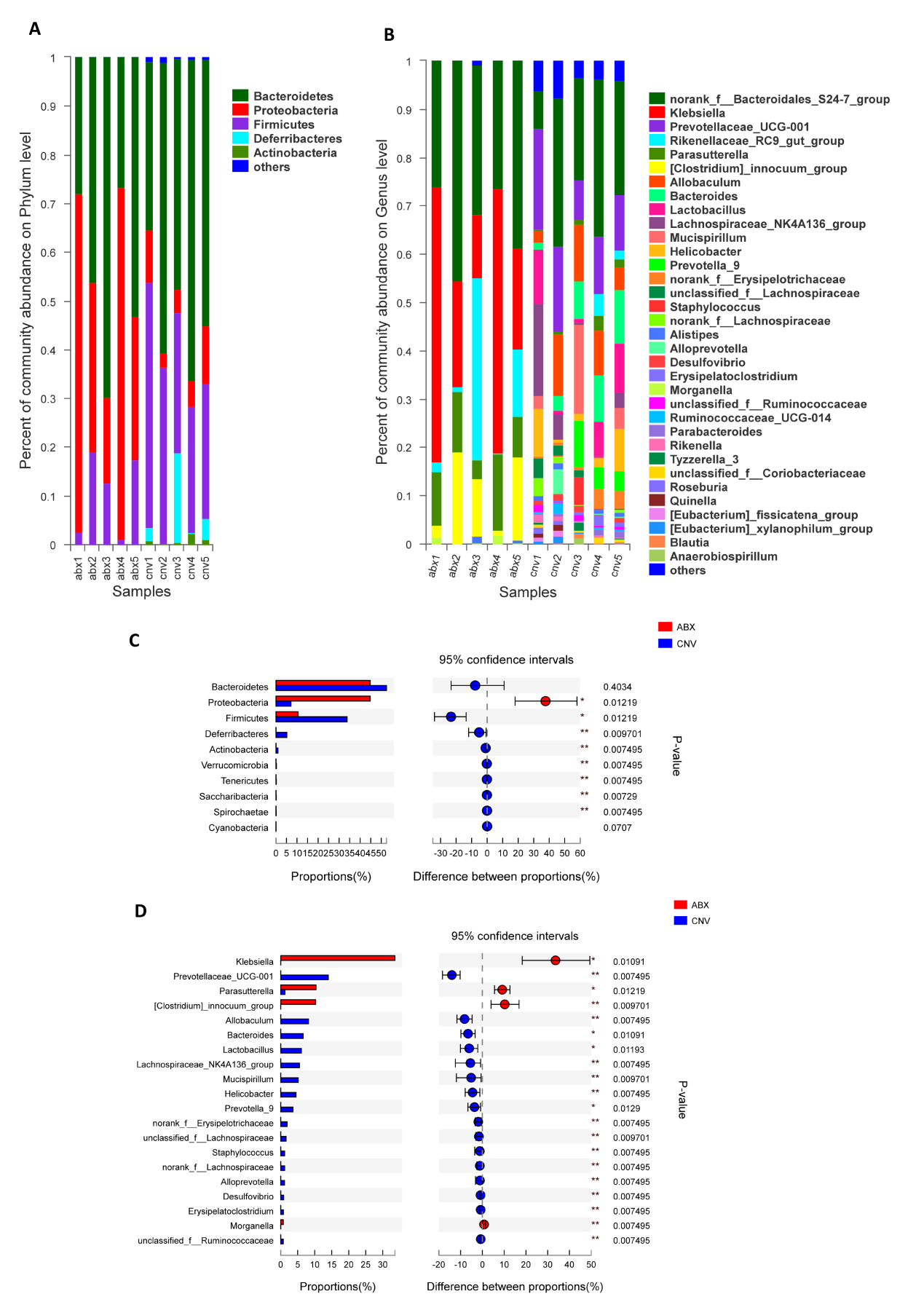

Supplement: Supplementary file 7 — Additional file 7: Figure S7. Community bar-plot and Wilcoxon rank-sum test bar plot. (A-B) Community bar-plot analysis shows relative abundance of intestinal microbiota in each sample at the phylum level (A) and genus level (B). (C-D) Wilcoxon rank-sum test bar plot on the phylum level (C) and genus level (D) showed significant genus in the top 20 of the total abundance at the classification level (n = 5, in each group). [file 13054_2020_2977_MOESM7_ESM.docx]
