# Supplementary material for: Microbiota-driven interleukin-17 production provides immune protection against invasive candidiasis
Source: Crit Care. 2020 May 27;24:268. doi: 10.1186/s13054-020-02977-5 (PMC7251893; doi:10.1186/s13054-020-02977-5)
Supplement: Supplementary file 2 [file 13054_2020_2977_MOESM1_ESM.docx]

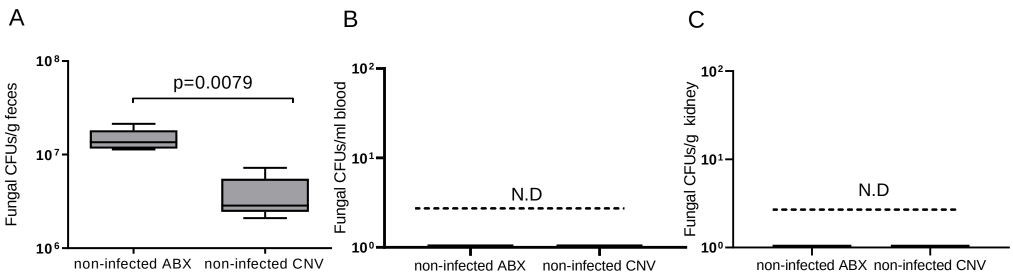
A
Fungal CFUs/g feces
10⁸
10⁷
10⁶
p=0.0079
non-infected ABX
non-infected CNV
B
Fungal CFUs/ml blood
10²
10¹
10⁰
N.D
non-infected ABX
non-infected CNV
C
Fungal CFUs/g kidney
10²
10¹
10⁰
N.D
non-infected ABX
non-infected CNV
