# Supplementary material for: Microbiota-driven interleukin-17 production provides immune protection against invasive candidiasis
Source: Crit Care. 2020 May 27;24:268. doi: 10.1186/s13054-020-02977-5 (PMC7251893; doi:10.1186/s13054-020-02977-5)
Supplement: Supplementary file 3 [file 13054_2020_2977_MOESM2_ESM.docx]

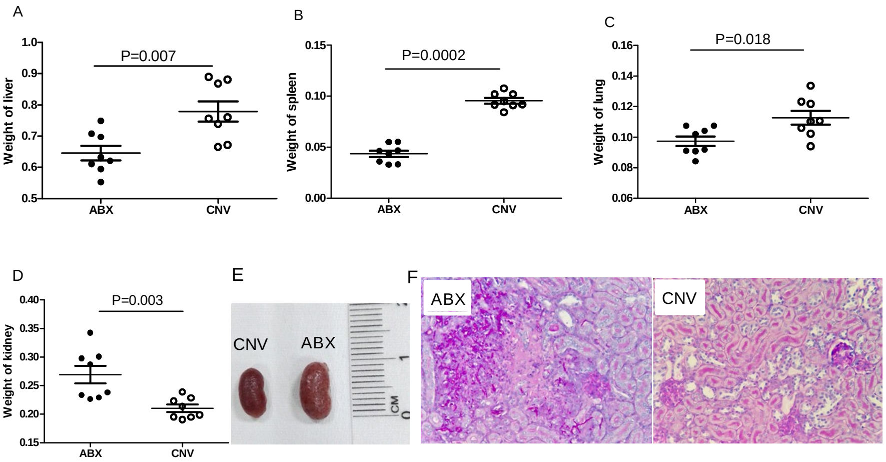
A
P=0.007
Weight of liver
1.0
0.9
0.8
0.7
0.6
0.5
ABX
CNV
B
P=0.0002
Weight of spleen
0.15
0.10
0.05
0.00
ABX
CNV
C
P=0.018
Weight of lung
0.16
0.14
0.12
0.10
0.08
0.06
ABX
CNV
D
P=0.003
Weight of kidney
0.40
0.35
0.30
0.25
0.20
0.15
ABX
CNV
E
CNV
ABX
F
ABX
CNV
