# Supplementary material for: Microbiota-driven interleukin-17 production provides immune protection against invasive candidiasis
Source: Crit Care. 2020 May 27;24:268. doi: 10.1186/s13054-020-02977-5 (PMC7251893; doi:10.1186/s13054-020-02977-5)
Supplement: Supplementary file 4 [file 13054_2020_2977_MOESM3_ESM.docx]

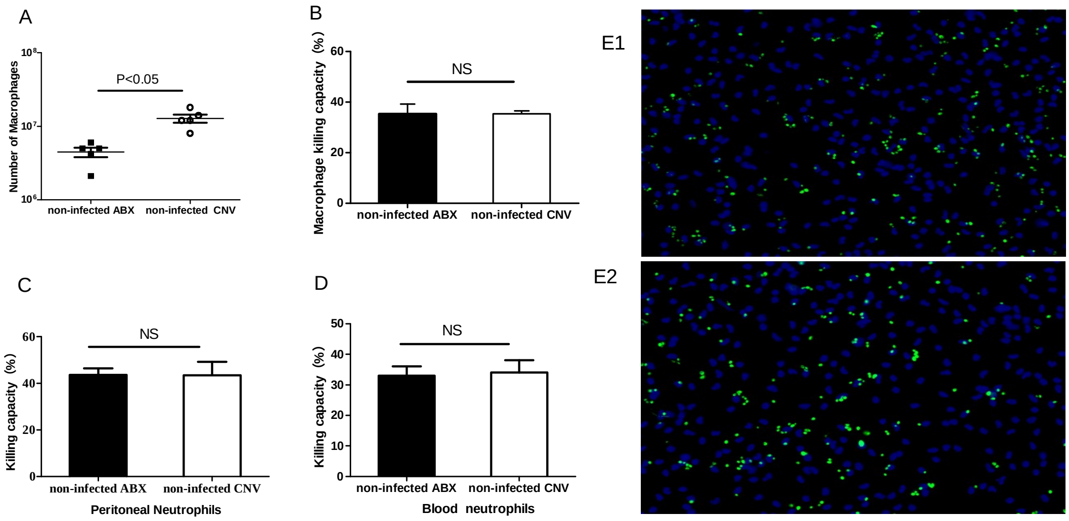
A
10⁸
10⁷
10⁶
Number of Macrophages
P<0.05
non-infected ABX
non-infected CNV
B
Macrophage killing capacity (%)
60
40
20
0
NS
non-infected ABX
non-infected CNV
E1
C
Killing capacity (%)
60
40
20
0
NS
non-infected ABX
non-infected CNV
Peritoneal Neutrophils
D
Killing capacity (%)
50
40
30
20
10
0
NS
non-infected ABX
non-infected CNV
Blood neutrophils
E2
