# Supplementary material for: Microbiota-driven interleukin-17 production provides immune protection against invasive candidiasis
Source: Crit Care. 2020 May 27;24:268. doi: 10.1186/s13054-020-02977-5 (PMC7251893; doi:10.1186/s13054-020-02977-5)
Supplement: Supplementary file 5 [file 13054_2020_2977_MOESM4_ESM.docx]

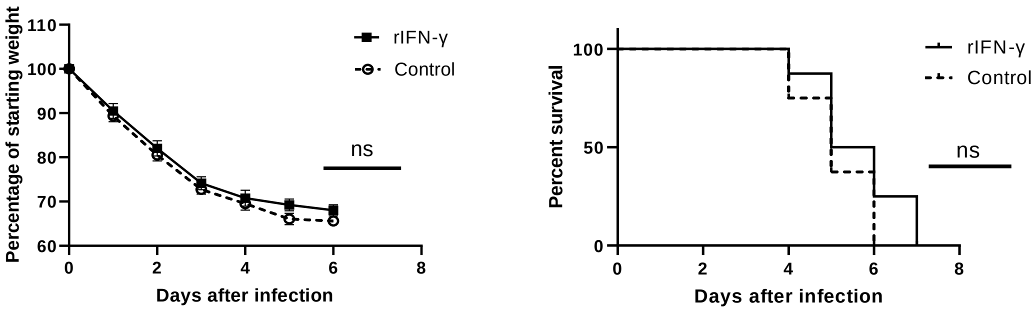
Percentage of starting weight
110
100
90
80
70
60
0
2
4
6
8
Days after infection
rIFN-γ
Control
ns
Percent survival
100
50
0
0
2
4
6
8
Days after infection
rIFN-γ
Control
ns
