# Supplementary material for: Microbiota-driven interleukin-17 production provides immune protection against invasive candidiasis
Source: Crit Care. 2020 May 27;24:268. doi: 10.1186/s13054-020-02977-5 (PMC7251893; doi:10.1186/s13054-020-02977-5)
Supplement: Supplementary file 6 [file 13054_2020_2977_MOESM5_ESM.docx]

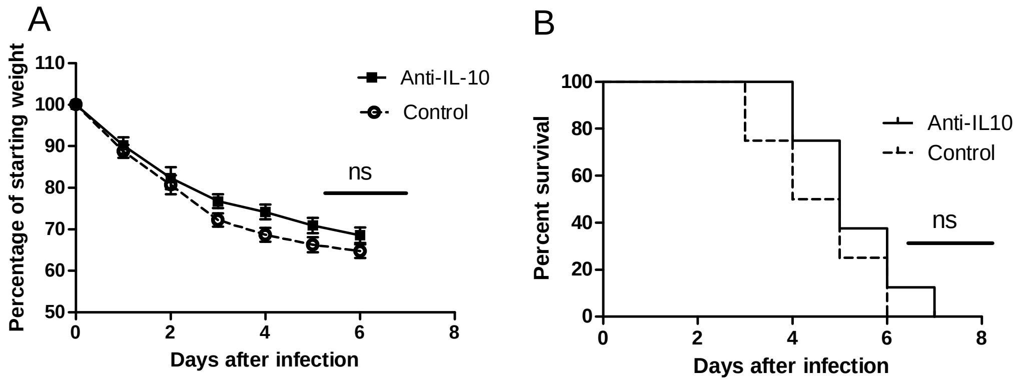
A
Percentage of starting weight
110
100
90
80
70
60
50
0
2
4
6
8
Days after infection
Anti-IL-10
Control
ns
B
Percent survival
100
80
60
40
20
0
0
2
4
6
8
Days after infection
Anti-IL10
Control
ns
